# Endometrial fluid biomarkers and their potential as predictors of successful embryo implantation

**DOI:** 10.37796/2211-8039.1413

**Published:** 2023-09-01

**Authors:** Ana R. Alves, Margarida F. Dias, Margarida Silvestre

**Affiliations:** a Faculty of Medicine, University of Coimbra, Portugal; b Gynecology Clinic, Faculty of Medicine, University of Coimbra, Gynecology Department, Hospital and University Centre of Coimbra, Portugal; c Bioethics Institute, Faculty of Medicine, University of Coimbra, Portugal

**Keywords:** Infertility, Endometrium, Embryo implantation, Biomarkers, Reproductive techniques, Assisted

## Abstract

**Background:**

Embryo implantation is a complex biological process which requires synchronized dialogue between the receptive endometrium and the blastocyst. The endometrium, however, is only receptive to embryo implantation for a very short period. Recurrent implantation failure (RIF) is a major challenge in assisted reproductive techniques mainly due to impaired receptivity, but there is still a need for a reliable and valid clinical test to assess endometrial receptiveness, especially at embryo transfer time. The aim of this review is to investigate what is currently known about the contribution of endometrial fluid (EF) to endometrial receptivity by identifying its potential biomarkers.

**Methods:**

This study involved an extensive search of the electronic databases PubMed and Cochrane, covering the period from 2011 to 2022. A combination of Medical Subject Headings with the terms ‘endometrial fluid’ and ‘embryo implantation’ was used.

**Results:**

Several different proteins presented in the endometrial cavity fluid have been described but the most consistent as potential biomarkers were Proprotein Convertase 6 (PC6), Vascular Endothelial Growth Factor (VEGF), PIGF (Placental growth factor), β3 integrin, Colony Stimulating Factor-3 (CSF-3), Leukaemia inhibitory factor (LIF), glycodelin and extracellular vesicles (EVs).

**Conclusions:**

Strong indicators support the use of uterine fluid collection as a non-invasive tool for receptivity assessment. Therefore, it could improve outcomes of assisted reproductive techniques.

## 1. Background

Embryo implantation is a complex biological process which requires synchronized dialogue between a functional embryo (blastocyst) and a receptive endometrium. The endometrium only becomes receptive for a limited period, during the mid-secretory phase of the menstrual cycle (days 20–24 of the cycle), known as the window of implantation (WOI) [1]. During this time of maximum receptivity, the embryo–endometrium interaction is optimal, beginning with apposition and culminating in the adhesion and invasion of the embryo into the endometrial epithelium [1].

In the endometrial tissue, under rising levels of progesterone, there is an increase in glandular secretion which is crucial for the synthesis and transport of mediators into the endometrial cavity, providing support and modulating the embryonic implantation [2]. The implantation process involves apical bonding and adhesion of the trophoblast cells to the endometrial epithelium. This process is only possible in this period and is associated with a loss of microvilli and a decrease in the amount of glycocalyx [3]. Nidation represents a critical event in the establishment of pregnancy and formation of an efficient placenta is dependent on it. Due to inadequate uterine receptivity, the entire process is compromised, resulting in failure of implantation or inadequate implantation of the embryo. This is considered a limiting factor of success of assisted reproductive techniques (ART) [4], widely used in the treatment of infertility. This complex health problem has wide clinical, psychological and socioeconomic repercussions and is currently on the increase: it affects about 8%–12% of reproductive age couples worldwide [5].

In view of this and despite the many advances in techniques, ART success rates remain low. European data in 2017 showed clinical pregnancy rates after in vitro fertilization (IVF) of 26.8% (aspiration) and 34.6% (transfer) and after intracytoplasmic injection (ICSI) of 24% (aspiration) and 33.5% (transfer). When freeze all cycles were removed, the clinical pregnancies per aspiration were 30.8% (IVF) and 27.5% (ICSI) [6].

Approximately 10% of women experience recurrent implantation failure (RIF) after in vitro fertilization-embryo transfers [7].

RIF, defined as the inability to establish a pregnancy after three full cycles of IVF, results from embryonic and/or uterine anomalies. Embryonic causes include anomalies in the oocyte/sperm quality and embryonic chromosomal, genetic and metabolic abnormalities. Uterine conditions consist of impaired endometrial receptivity, local immune disorders at the site of implantation and some gynecological pathologies such as endometriosis or adenomyosis, tubal pathology, uterine fibroids or endometrial polyps. Even the IVF procedure itself may have a negative influence [8].

The progress in embryo selection, in particular through the use of pre-implantation genetic screening, allows for the transfer of high-quality embryos, so that compromised endometrial receptivity plays a decisive role in situations of implantation failure.

Some studies have considered endometrial receptivity as accounting for two-thirds of implantation failure and the embryo for only one-third of cases [4,9–11].

Therefore, in cases of RIF of unexplained cause which have favorable hormonal response, no known gynecological pathology, high embryo quality and satisfactory endometrial development, suboptimal endometrial receptivity is considered the key factor [4,7]. Although it represents a main challenge for contemporary reproductive medicine, implantation remains insufficiently characterized, mainly due to the lack of reliable biochemical markers that allow assessment of endometrial receptivity [7].

Faced with this need, endometrial fluid has been recently proposed as a potential agent. This article reviews current literature about endometrial receptivity biomarkers present in endometrial fluid.

## 2. Materials and methods

A literature search was performed on the PubMed and Cochrane for all published articles between 2011 and 2022. The literature search was conducted using the combination of the following terms *(‘endometrial fluid’* or *‘uterine fluid’* or *‘uterine flushing’* or *‘endometrial fluid collection’)* or *(‘e*xtracellular vesicles’ or ‘PC6'or ‘VEGF’ or ‘PIGF’ or ‘β3 integrin’ or ‘CSF-3'or ‘LIF’ or ‘glycodelin'or ‘extracellular vesicles’) and (‘endometrial receptivity’ or *‘Assisted Reproductive Techniques’* or *‘infertility’* or *‘embryo implantation’* or ‘embryo development’ or ‘endometrium’ or *‘recurrent implantation failure’* or *‘repeated implantation failure’* or *‘biomarkers’*). The search was performed in English. Both animal and human studies were included for this review. In addition, all relevant studies were identified, included and the results were summarized. A total of 411 articles were reviewed.

### 2.1. Endometrial fluid

Endometrial fluid is a complex biological liquid, in direct contact with the endometrial cavity, which contains multiple molecules secreted from the endometrium [12,13]. Although its exact constitution is not fully understood, it includes biologically active molecules such as amino acids, steroids, glucose, lipids, ions, transport proteins, cytokines, hormones, enzymes, growth factors, proteases, inhibitors and immunomodulators [14].

Its components result from the selective transudation of the serum (90% of the total proteins) from the peritoneal, ovarian and uterine tube fluids; leukocyte release from the uterine cavity and products resulting from the cleavage of surface proteins and glycocalyx (mucin-rich layer that covers the apical cell surface of the endometrium), as well as endometrial glandular secretion [14].

The main roles of the endometrial fluid in the uterine microenvironment are to provide support, immunosuppression and to modulate certain functions of the blastocyst (uterine fluid provides essential nutrients and information for blastocyst development) relevant to endometrial-embryo crosstalk during the pre-implantation period, representing the interface between the floating embryo and the endometrium, allowing the transfer of vital information) [15,16].

Uterine fluid most likely has a different composition in conception and non-conception cycles [17]. Some evidence suggests that endometrial fluid during the non-implantation cycle increases inflammation, as it contains inflammatory mediators and consequently alter endometrial receptivity [18,19].

The importance of this microenvironment goes far beyond implantation, providing nutrition to the embryo and shaping the programming of its development - EF may have adverse effects on cell proliferation or interfere with the early stages of embryo implantation [19,20].

Blood flow through the placenta is only established at the end of the first trimester of gestation; until then, the embryo is dependent on bioactive mediators which are produced by uterine glands, present in the fluid.

Another potential non-molecular role is regulation of the movement of the embryo within the uterine cavity during the middle secretory phase, reaching a maximum of ~50 μl [14].

The existence of an excessive amount of fluid in the endometrial cavity (defined by an increase of >3.5 mm in anterior-posterior diameter between adjacent endometrial surfaces identified during transvaginal ultrasound) compromises implantation because of its biochemical effects - direct action on embryonic development (embryotoxicity),due to the inflammatory nature of the fluid and mechanical effects (flushing the cavity - compromised endometrial receptivity, embryonic apposition and discharge of the embryo) [21,22].

The presence of EF during controlled ovarian hyperstimulation (COH) has been associated with tubal factor infertility (with and without hydrosalpinges), polycystic ovarian syndrome (PCOS), subclinical uterine infections, severe endometriosis and previous uterine surgery [19].

Recent studies reported that the presence of endometrial cavity fluid (even when the quantity is less than 3.5 mm) is an indicator of adverse pregnancy outcomes (implantation does not occur due to flushing of the cavity or abnormal endometrial receptivity) and an indication to freeze all embryos [18,19].

Current knowledge is limited, other studies reported that pregnancy rates remained stable when EF was detected in small amounts (<3 mm) and is absent on the day of embryo fresh transfer. Therefore, it appears that both the quantity and timing of EF appearance are important factors to consider [19].

Faced with an RIF, the study of the embryo has been given priority, with consequent optimization of its selection and transfer.

With the advance of molecular biological technologies, approximately 1500 proteins present in the uterine fluid collected from fertile and primary infertile women were identified in the proliferative phase [20,21,23].

### 2.2. Potential biomarkers

#### 2.2.1. Proprotein Convertase 6 (PC6)

Proprotein convertase 6 (PC6) is an important enzyme for activation of inactive protein precursors [24]. It is expressed in the endometrium, especially in the most receptive phase, with a preponderant role in embryo implantation. It is also present in endometrial fluid, where its concentrations are also related to the state of receptivity, being higher in the receptive phase compared to non-receptive when in fertile women and markedly reduced during the implantation window in infertile women [25].

PC6 cleaves the protein phospholipid Ezrin-Radixin-Moesin-Binding Phosphoprotein 50 (EBP50) affecting its interaction with the protein ezrin, which in turn regulates the interface between the cytoskeletal actin and the plasma membrane, with redistribution along the apical membrane to the cytoplasm leading to a reorganization of the cytoskeleton. The apical location of these proteins contributes to the formation of microvilli and cell polarization; its non-adherent character also influences the morphology of endometrial epithelial cells and embryo adhesion during implantation. In the absence of ezrin, the microvilli are short, thick and randomly oriented rather than tall, uniform and densely clustered [26]. Endometrial PC6 is an important regulatory factor in the activation of platelet-derived growth factor type A (PDGFA). In the proliferative phase the levels of PDGFA were very low, unlike those observed in the middle secretory phase, where activated PDGFA was clearly detected on the apical surface of the luminal and glandular epithelia. Non-activation by PC6 implies that PDGFA does not bind to its receptors and may compromise receptivity [24]. The function of alpha-dystroglycan (α-DG) in the adhesion process depends on the removal of its terminal part, α-DG-N, by PC6. When non-removed there is a retention of the α-DG on the cell surface, with loss of adhesiveness, thus constituting a barrier to implantation [27]. The amount of α-DG-N in the uterine fluid may reflect its removal from the endometrial tissue and intuit the state of receptivity.

#### 2.2.2. Vascular Endothelial Growth Factor (VEGF), placental growth factor (PIGF) and soluble fms-like tyrosine kinase (sFlt-1)

The success of embryo implantation depends on coordinated vascular development and subsequent maintenance of the maternal uterus–embryo interface, in order to provide adequate nutrition.

Physiological angiogenesis usually does not occur in most organs, except when they are injured. The female reproductive system is an exception, with fundamental angiogenesis during the menstrual cycle and implantation [28].

Vascular endothelial growth factor (VEGF), produced by the glandular epithelium and abundant in the uterine fluid, is a potent angiogenesis factor [5], recognized as a vascular endothelial cell growth factor involved in carcinogenesis [13], vessel growth and remodeling of tissues, such as the endometrium. It contributes to the adhesion between endometrial epithelium and extracellular matrix of the trophoblast and also to the blastocyst development [5,29].

Studies have shown significantly reduced levels of VEGF during the middle secretory phase in women with unexplained infertility, when compared to fertile women [13,30]. In turn, VEGF levels in fertile women are higher in the secretory phase than in the proliferative phase [29].

Placental growth factor (PIGF) is a homologous molecule of VEGF, present in the endometrial fluid, which binds exclusively to the VEGF receptor with a higher affinity than VEGF itself. The PIGF acts on blastocyst cell number and outgrowth and is highly expressed in the placental trophoblast in all stages of pregnancy. It acts on growth control, differentiation and invasion of the trophoblast in the maternal decidua. In addition, it has effects on the endometrial epithelium and its adhesive capacity, promotes endothelial cell mitosis and increases chemotaxis for leukocytes. PIGF is predominantly located in the luminal/glandular epithelial cells of the endometrium throughout the menstrual cycle (fertile and infertile group), as well as in the cells surrounding the maternal spiral arteries during the secretory phase. Its location varies according to the phase of the cycle: during the early secretory phase it is located in basal layer of the epithelial cells and in the late stage it is located in apical region, being released into uterine secretions [2]. It is also detectable in maternal serum, which allows preeclampsia to be signaled when there is an early and marked decrease in its serum levels [2].

Recent studies have shown elevated concentrations of PIGF, sFlt-1, sGP130, IL-8 and CSF-3, but not of VEGF or Fibroblast Growth Factor 2 (FGF-2), in the middle secretory phase in endometrial fluid of women with idiopathic primary infertility. High concentrations of PIGF may be associated with infertility, inducing a possible false state of pregnancy in the endometrium, preventing the receptivity and fixation of the embryo [31].

Soluble fms-like tyrosine kinase (sFlt-1) is a soluble antagonist of VEGF and PIGF which increase in the proliferative phase and decrease during the luteal phase. Infertile women had higher serum sFlt-1 levels in the secretory phase than in the proliferative phase during decidualization (as opposed to VEGF).

#### 2.2.3. Integrin β3

Integrins are a group of cell adhesion molecules which are anchored to the cytoplasmic membrane.

Integrin ανβ3 is secreted by the endometrial epithelium during the middle secretory phase and is involved in the initial phase of blastocyst implantation [32]. A reduction in its expression during the luteal phase is associated with idiopathic infertility and recurrent failure of embryo transfer in IVF cycles [33]. Conditions associated with subfertility such as endometriosis, hydrosalpinx and luteal phase insufficiency have also been associated with altered expression of integrins [32].

Current evidence is controversial; the pattern of differential expression among fertile and infertile women has not been established and the expression of the αvβ3 integrin in the endometrium has reduced reproducibility and high variability [33].

#### 2.2.4. Colony stimulating Factor-3 (CSF-3)

The family of colony stimulating factors includes: macrophage CSF (M-CSF), granulocyte macrophage (GMCSF) and granulocyte CSF (G-CSF). Each one has a certain expression in the reproductive system, acting in ovulation, embryo implantation, embryo and placental development, due to its immunomodulatory, immunotropic and anti-apoptotic properties during the beginning of the pregnancy [34].

They are used in current practice as oocyte quality biomarkers (follicular G-CSF), embryonic culture medium supplements (recombinant human GMCSF) and as innovative therapy in infertility (human recombinant GCSF) [34]. G-CSF is a cytokine that regulates production, proliferation and migration of neutrophils, modulation of inflammatory response, promotion of granulocytic cell survival and proliferation of trophoblast cells. It also contributes to the regulation of the cytotoxicity of uterine NK cells, reducing the production of interferon and *IL18* [34].

Its receptors are expressed in the placenta, endothelial cells, cells of the central nervous system and cardiomyocytes. Macrophages and other specialized cells produce inflammatory molecules (endotoxins and cytokines) that stimulate transcription mechanisms implying changes in CSF-3 and a consequent recruitment and activation of neutrophils [35].

Recent studies established that the endometrium is exposed to high levels of G-CSF during almost all the menstrual cycle, as well as the arrival at the blastocyst in the uterine cavity. This was the only significantly increased marker in idiopathic infertile women during the early secretory phase [31].

#### 2.2.5. Leukaemia inhibitory factor (LIF)

Leukaemia inhibitory factor (LIF) is a pleotropic cytokine, expressed in the endometrium, blastocyst and placenta. It regulates implantation through the transformation of the endometrium into a receptive state and the adhesion of the blastocyst (promoting the development of pinopodes) to the endometrial epithelium [5], promoting stromal decidualization, trophoblast invasion, blastocyst development and leukocyte uterine infiltration [36].

In the endometrium, LIF is scarce during the proliferative phase and increase after ovulation. The level remains elevated until the end of the cycle, reaching maximum expression during the middle secretory phase and becomes detectable in the uterine fluid [37,38]. LIF is apparently independent of the embryo and relatively dependent on maternal sex hormone levels [38].

Recent studies indicate a reduction in LIF levels in the endometrium and uterine fluid during the secretory phase in women with recurrent implantation failure [5,16,30,38]. LIF levels in uterine fluid were all significantly higher in fertile women and pregnant women as compared with nonpregnant women [30].

Concentrations obtained from the endometrial fluid of infertile women were lower compared to controls, indicating that a deregulation in its production may justify a decrease in uterine receptivity, leading to RIF and abortion [39]. In the same way, LIF levels were significantly lower in the uterine fluid of patients with adenomyosis [37]. In serum it does not reflect the state of fertility [9]; however, the reduced concentrations in the uterine fluid seems to be predictive of an unsuccessful implantation [39].

#### 2.2.6. Glycodelin

Glycodelin is a glycoprotein mainly produced by the endometrial glandular epithelium (during the luteal phase, following progesterone secretion) [5] and to a lesser extent by extrauterine tissues, tubes, ovaries, hematopoietic cells and mammary gland. It is also expressed in pregnancy decidua and amniotic fluid [40,41].

This glycoprotein promotes the existence of the implantation window by suppressing the maternal immune response to the embryo [5], through the inhibition of T lymphocyte proliferation cytokine secretion and modulation of NK-cell cytokine production [42]. However, it does not cause detectable generalized immunosuppression because the blood concentration is too low [41].

Furthermore, it participates in cancer development, being specifically expressed in some malignant cells such as breast, ovary, endometrium, colon and lung cancers [41].

Plasma glycodelin increases five days after the peak of LH and returns to baseline during the following follicular phase. In conception cycles, it increases rapidly after implantation, peaking at 8–10 weeks of gestation and then decreasing. It is diminished in pregnant women with ovarian failure [41], after treatment with gonadotropin - releasing hormone agonist (GnRH-a) and after clomiphene citrate stimulation, which represents an altered endometrial function in pregnancies after ovarian stimulation [40]. Still, plasma glycodelin is elevated in endometrial cancer patients compared to the control group. Therefore, it could be a potential biomarker for early diagnosis and recurrence monitoring and a potential target for cancer immunotherapy [41].

In uterine flushing it is detectable from the 6th post-peak day of LH and increases fast. In the late luteal phase, it exceeds plasma concentrations 100 times[40]. Glycodelin fluid levels present a positive correlation with the state of endometrial maturation [16,40]. The concentration in the serum and endometrial fluid increases in the ovulatory cycles [43] and a decrease in the fluid of infertile women can be seen [40]. In early pregnancy, the maximum peak in serum and amniotic fluid occurs at 6–12 weeks of pregnancy [42].

#### 2.2.7. Extracellular vesicles

Extracellular vesicles (EVs) are cell-derived membranous vesicles that transport bioactive molecules and that are found in a vast variety of biological fluids (plasma, semen, uterine fluid) and tissues (liver, uterus, embryo, cultured endometrial epithelial/stromal cells, trophectodermal). These are emerging as one of the critical components in the effective communication between the receptive endometrium and the blastocyst, through the release of secreted factors within the endometrial fluid [44]. EVs secreted by endometrial cells are taken up by embryonic cells and carry proteins that improve the quality of the embryo and regulate its implantation, as well as modulate the adhesive capacity of the trophoblast. Within the uterine cavity, these molecules contribute to maintain a partially immune-suppressive local microenvironment in the embryo-maternal crosstalk [44].

Its protein composition is dynamically regulated during the menstrual cycle. During the pre-implantation phase, EVs in the uterine fluid containing a greater abundance of proteins involved in cell apoptosis whereas during implantation, they mainly contain proteins involved in cell adhesion. The highest concentration occurs in the luteal phase of the menstrual cycle [45,46].

In recurrent implantation failure (RIF), endometrial EVs inhibit embryogenesis (inhibit blastocyst formation, depletion of the number of embryo cells) and implantation potential (decreasing trophoblast cell proliferation, migration and invasion) [46].

EVs can predict the implantation window, thus highlighting its potential as a minimally invasive biomarker allowing the diagnosis of impaired implantation and the optimal timing for embryo transfer [40].

Extracellular vesicles derived from endometrial cells from uterine lavages may represent a sample of potential molecular markers for monitoring endometrial status [47].

## 3. Conclusions

Embryo implantation remains a challenge in reproductive medicine. The identification of reliable biomarkers of endometrial receptivity may represent an increase in the efficacy of IVF treatments by optimizing the costeffectiveness of treatment and increasing pregnancy rates.

Analysis of uterine fluid, collected by lavage or aspiration, is a less invasive approach than a biopsy, requiring only the insertion of an embryo transfer catheter for fluid aspiration [48].

It can be performed prior to embryo transfer in the same cycle, without any adverse effect on pregnancy rates (does not affect embryo implantation rate) [49,50].

Indeed, based on data discussed, endometrial fluid may represent a potential non-invasive biomarker that can be analyzed during the period of endometrial receptivity [50].

However, although endometrial fluid analysis provides instantaneous information of the uterine environment during the implantation window, understanding of the specific glandular contribution to the total human uterine fluid composition remains limited. Despite the many advances in research, some issues remain unclear: a) the variability of the beginning of receptivity period within the same woman and in different women b) if all women reach uterine receptivity in all cycles; c) the possibility of biomarkers being diverse in women with different etiologies for infertility; d) which markers of normal receptivity are altered in women whose infertility is due to impaired receptivity; e) if a receptive endometrium, when receiving a good quality embryo, always results in a pregnancy [17,51].

So, there are few transcriptional studies related to endometrial receptive markers from endometrial fluid. Current valid markers used to assess the endometrial receptivity during the optimal WOI are not established, mainly due to methodological heterogeneity and inconsistent results of the reviewed studies, insufficient data and lack of validation. Furthermore, proteomic approaches are technically complex and time-consuming for the integration into clinical practice [52].

Therefore, despite the progress made in understanding the physiology and pathophysiology of the human endometrium, there is a need for multiple simpler and more effective modalities for a comprehensive assessment of endometrial state during WOI.

## List of abbreviations

ART: Assisted Reproductive Techniques
α-DG: Alpha-Dystroglycan
COH: Controlled Ovarian Hyperstimulation
CSF-3: Colony Stimulating Factor-3
EBP50: Ezrin-Radixin-Moesin-Binding Phosphoprotein 50
EF: Endometrial Fluid
EVs: Extracellular Vesicles
FGF2: Fibroblast Growth Factor 2
G-CSF: Granulocyte CSF
GM-CSF: Granulocyte Macrophage
GnRH-a: Gonadotropin Releasing Hormone agonist
ICSI: Intracytoplasmic injection
IL: Interleukin
IV,F: In Vitro Fertilization
LH: Luteinizing Hormone
LIF: Leukaemia Inhibitory Factor
M-CSF: Macrophage CSF
NK: Natural Killer
PCOS: Polycystic Ovarian Syndrome
PC6: Proprotein Convertase 6
PIGF: Placental Growth Factor
PDGFA: Platelet Derived Growth Factor Type A
RIF: Recurrent Implantation Failure
sFlt-1: Soluble Fms-like tyrosine kinase
VEGF: Vascular Endothelial Growth Factor
WOI: Window Of Implantation
